# Effects of Self-management Program as Adjunctive to Usual Rehabilitation Exercise on Pain and Functional Outcomes in Knee Osteoarthritis: A Randomized Controlled Trial

**DOI:** 10.34172/jrhs.2023.104

**Published:** 2023-03-30

**Authors:** Mohd Azzuan Ahmad, Ashril Yusof, Mohamad Shariff A Hamid, Faizul Hafiz Zulkifli Amin, Siti Salwana Kamsan, D Maryama Ag Daud, Devinder Kaur Ajit Singh

**Affiliations:** ^1^Physiotherapy Program, Centre for Rehabilitation and Special Needs Studies, Faculty of Health Sciences, Universiti Kebangsaan Malaysia, Kuala Lumpur, Malaysia; ^2^Faculty of Sports and Exercise Science, Universiti Malaya, Kuala Lumpur, Malaysia; ^3^Sports Medicine Unit, Faculty of Medicine, Universiti Malaya Medical Centre, Kuala Lumpur, Malaysia; ^4^Department of Physical Rehabilitation Sciences, Faculty of Allied Health Sciences, International Islamic University Malaysia, Pahang, Malaysia; ^5^HEAL Research Unit, Faculty of Medicine and Health Science, Universiti Malaysia Sabah, Sabah, Malaysia; ^6^Centre for Healthy Ageing and Wellness, Faculty of Health Sciences, Universiti Kebangsaan Malaysia, Kuala Lumpur, Malaysia

**Keywords:** Exercise, Home-based, Knee osteoarthritis, Patient education, Self-management

## Abstract

**Background:** Home-based exercise (HBE) and patient education (EDU) have been reported as beneficial additions to usual knee osteoarthritis (KOA) rehabilitation. However, previous trials mostly examined the effects of HBE and EDU separately. Thus, this study aimed to evaluate the effects of a structured combined HBE and EDU program in addition to usual KOA rehabilitation on pain score, functional mobility, and disability level.

**Study Design:** A parallel-group, single-blinded randomized controlled trial.

**Methods:** Eighty adults with KOA were randomly allocated to experimental (n=40) and control (n=40) groups. All participants underwent their usual physiotherapy care weekly for eight weeks. The experimental group received a structured HBE+EDU program to their usual care, while the control group performed home stretching exercises to equate treatment time. The Knee Injury and Osteoarthritis Outcome Score (KOOS) for the disability level, visual analogue scale (VAS) for pain, and timed up-and-go test (TUG) for mobility were measured pre-post intervention.

**Results:** After eight weeks, the experimental group demonstrated significant improvements in the KOOS (all subscales), pain VAS, and TUG scores compared to baseline (*P*<0.001); meanwhile, only KOOS (activities of daily living and sports subscales) was significant in the control group. Relative to the control, the experimental group presented higher improvements (*P*<0.001) by 22.2%, 44.1%, and 15.7% for KOOS, pain VAS, and TUG, respectively.

**Conclusion:** Integrating the HBE+EDU program into usual KOA rehabilitation could reduce pain and disability, while it improved functional mobility. The finding of this study suggests a combination of a structured HBE and EDU program to be considered as part of mainstream KOA management.

## Background

 The incidence and prevalence of knee osteoarthritis (KOA), the most common form of arthritis^1^, is rapidly increasing.^1,2^ This progressive disease is commonly characterized by knee pain, limited knee movement, joint stiffness, muscle weakness, functional mobility impairments, and postural balance disturbances.^1^ If untreated, it could lead to permanent disabilities associated with deterioration in physical and mental health,^3,4^ which in turn affect the quality of life (QOL).^5^ The majority of KOA patients undergo supervised exercise therapy (SET),^6^ which has been shown effective in improving KOA symptoms^7,8^ and delaying disease progression.^1,4^ However, SET implementation in daily practice has its limitations; optimally, SET should be performed at least three times per week,^9^ which is possibly impracticable due to scheduling limitations (time and logistics),^10^ as well as cost and healthcare resources.^11,12^ In addition, excessive reliance on healthcare services due to continuous SET resulted in a decline in patients’ self-management and awareness.^13,14^ Moreover, although SET is a mandatory conservative treatment, about 40% of KOA patients reported that their current rehabilitation program is ineffective,^5^ implying that SET alone is insufficient in KOA management.^1,9^ Hence, a more feasible and efficient long-term strategy is necessary for KOA, which can be achieved by switching from health-centered to self-centered management.^1,15^

 Among the self-management strategies in KOA are the implementations of HBE^16,17^ and EDU programs.^8,13,18^ The components of HBE are similar to those prescribed during SET; however, the exercises are modified to be independently performed at home without needing professional supervision or special equipment.^16^ For long-term management of KOA, HBE is effective in reducing pain,^8,16^ improving knee functions such as strength and flexibility,^17,19^ and decreasing the level of disability.^19,20^ Meanwhile, an EDU program includes information on disease processes, self-management skills, coping strategies, and guidance on a healthy lifestyle.^13,15,21^ These are mirrored in improved skills (managing pain and activity of daily living),^8,10^ self-confidence,^22,23^ self- management,^10^ and awareness of knee rehabilitation exercises.^13^ In contrast, a lack of knowledge among KOA patients has negatively impacted treatment outcomes.^15^ Based on this evidence, a combination of HBE and EDU would seem ideal for greater improvements in the self-centered management of KOA rehabilitation.

 A structured combination of HBE and EDU may exert greater benefits in KOA management^6,19^; however, previous trials mainly evaluated the effects of these adjunctive treatments separately,^8,10,17^ and little is known about the effects of the combination of these adjunctive treatments. Therefore, this study sought to investigate the outcome of integrating a home-based exercise and patient education program (HBE + EDU) in addition to usual physiotherapy care on pain, functional mobility, and disability level among adults with KOA. It was hypothesized that this form of treatment could improve pain, functional mobility, and disability among KOA patients. The findings of this study will allow healthcare practitioners to design an effective self-management program for optimal functional outcomes and reduce reliance on healthcare services.

## Methods

###  Study design

 This study was a parallel-group, single-blinded (outcomes assessor), randomized controlled trial. The study was approved by the Secretariat for Research and Ethics of Universiti Kebangsaan Malaysia (UKM PPI/111/8/JEP-2017-021) and Medical Research Ethics Committee of Universiti Malaya Medical Centre (MREC ID: 2020102-9129) in compliance with the Declaration of Helsinki (1975). The study protocol was designed according to the Consolidated Standards of Reporting Trials (CONSORT) guidelines. This trial was registered with the Australian New Zealand Clinical Trials Registry (ACTRN12618001434280).

###  Study setting and population

 This study was conducted at the Universiti Kebangsaan Malaysia and Universiti Malaya Medical Centre, Malaysia. Adults with symptomatic (knee pain or stiffness) KOA were screened and recruited from the Physiotherapy Department of Universiti Kebangsaan Malaysia and Universiti Malaya Medical Centre. Patients who fulfilled the inclusion and exclusion criteria were invited to participate in this study. The inclusion criteria were adults aged 18 years old and above with symptomatic KOA, clinical or radiographic diagnosis of KOA based on the American College of Rheumatology criteria, and ability to walk independently for at least 3 m. However, the exclusion criteria were inflammatory arthritis (e.g., rheumatoid arthritis, psoriatic arthritis, ankylosing spondylitis, and systemic lupus erythematosus), acute traumatic knee injury (ligamentous or meniscal tear), and current participation in a structured exercise program or interventional study.

###  Sample size calculation

 The required sample size to detect a difference between the means of the two groups was estimated using the G*Power statistical analysis software, version 3.1.9.7.^24^ The parameters applied in the calculation were based on previous studies, including a two-sided alternative test, an effect size (ES) of 0.2, an alpha level of 5%, and pre-specified statistical power of 80%.^24,25^ The estimated total sample size was 52 participants. However, about 40 participants per group were determined considering a 50% dropout rate.

###  Sampling and randomization

 The process of screening, recruitment, and randomization (group allocation) of participants was performed by a researcher who was not involved in the intervention or outcome assessment. Eligible participants (n = 80) were randomized in a 1:1 allocation ratio to one of the two intervention groups (experimental or control) using a computer-generated randomization table. The allocation concealment was achieved using a sealed opaque envelope describing the treatment group. All participants had to sign an informed consent form after receiving verbal and written information on the study protocol.

###  Procedures

 In this study, both groups received their usual physiotherapy care weekly for eight weeks. The usual physiotherapy treatment was prescribed and tailored to the individual participants’ needs based on recommended KOA treatment guidelines.^9,26^ The treatment was administered by qualified physiotherapists who were blinded toward the participants’ group allocation. The usual physiotherapy treatment consisted of physical modalities (e.g., hot packs, cryotherapy, ultrasound, and electrical stimulation) and therapeutic exercise, including manual therapy techniques (soft tissue mobilization and manipulation), mobilizing, stretching, strengthening, balance, proprioception exercise, and functional exercises.

 Participants in the experimental group received a combination of HBE + EDU program in addition to their usual physiotherapy care. The HBE was explicitly designed by adapting the contents from evidence-based treatment guidelines and relevant KOA-related studies.^13,17,27^ The HBE booklet, which comprises general information on KOA, exercise training guidelines, and daily exercise logs, was given to each participant in the experimental group. Participants were instructed to perform the HBE at least four times a week. The HBE was delivered in written and illustrated step-by-step images that consisted of mobilizing, stretching, strengthening, and functional exercises such as calf stretching, straight leg raise, static quadriceps, terminal knee extension, and sit-to-stand exercises. Meanwhile, EDU was delivered through a group session. One of the researchers provided this EDU session once for each participant in the first week of the intervention. The EDU was based on the HBE booklet, which covers KOA self-management and the importance of the exercise. The components of HBE + EDU are summarized in Table 1.

**Table 1:** HBE and Patient EDU program

**Components**	**Duration (min)**
HBE	30
(1) Range of motion: Prone knee bend and supine alternate knee bend (2 items, each item; 10 repetitions, 2 sets)	5
(2) Stretching: Standing quadriceps stretch, calf stretch in long sitting, and supine hamstring stretch (3 items, each item; 15-second hold, 3-5 repetitions, 1 set)	5
(3) Strengthening: Sitting knee extension, supine straight leg raise, static quadriceps, and side-lying straight leg raise (4 items, each item progression: Week 1-2 (5-second hold, 5 repetitions, 1 set), week 3-5 (5-10-second hold, 7-10 repetitions, 1 set), week 6-8 (10-second hold, 10 repetitions, 2 sets))	15
(4) Functional training: Sitting to stand 10 repetitions, standing mini-squat 10 repetitions, walking exercise for approximately 2 minutes (3 items; each item for approximately 2 minutes)	5
Patient EDU	30
Included components were a brief explanation of knee osteoarthritis (definition, signs and symptoms, risk factors, complications, and the recommended management), highlights on physiotherapy management, importance and benefits of exercise therapy, and general advise (e.g., dietary, self-management strategies for knee pain, and dos and don’ts).	

*Note*. HBE: Home-based exercise; EDU: Education.

 Meantime, to equalize treatment time between groups, participants in the control were instructed to perform simple home stretching exercises daily,^17^ including standing quadriceps stretch, long sitting calf stretch, and supine hamstring stretch (each item; 15-second hold, ten reps, two sets). Hence, both groups spent a comparable amount of time performing the therapeutic exercises; the experimental (HBE + EDU; 25 minutes per session, four days a week, approximately 100 minutes per week) and control (home stretching; 15 minutes per session, daily, about 100 minutes per week) groups. Additionally, all participants were provided with a training diary^17^ and contacted by telephone once every two weeks to remind them to continue performing home exercises (HBE or home stretching) based on their instruction.^28,29^

###  Outcome measures

 The primary outcome was the Knee Injury and Osteoarthritis Outcome Score (KOOS). It is a self-administered questionnaire to evaluate participants’ views on their knees and associated problems with KOA.^30^ It has five related subscales, including pain, symptoms, activities of daily living (ADL), sport and recreation, and QOL.^30^ KOOS is a valid and reliable outcome measure in different patient populations with KOA.^30-32^ The Spearman’s correlation coefficients for construct validity ranged from moderate to high between the KOOS subscales and the Western Ontario and McMaster Universities Arthritis Index (WOMAC).^31^ In addition, excellent test-retest reliability was reported with an interclass correlation coefficient ranging from 0.91 to 0.99,^32^ and Cronbach’s alpha values varied from 0.84 to 0.91, 0.25 to 0.75, 0.94 to 0.96, 0.85 to 0.89, and 0.64 to 0.9 for pain, symptoms, ADL, sport and recreation, and QOL, respectively.^30^

 On the other hand, the secondary outcomes were the visual analogue scale (VAS) for pain and the timed up-and-go test (TUG). The pain VAS is a valid tool with excellent test-retest reliability (an intraclass correlation coefficient of 0.97) to evaluate chronic pain in clinical practice.^33^ Meanwhile, the TUG is a test to evaluate an individual’s functional mobility. The test is performed by asking the subject to rise from a sitting position, walk for three meters, turn around, walk back to the chair, and sit down.^34^ The time taken to complete the test was recorded, and a score of more than 14 seconds indicated a high risk of falls.^34^ All the outcomes were measured at baseline (week 0) and immediately following the completion of the intervention (week 8) by the same assessor, who was blinded to the group allocations.

###  Statistical analysis

 The data were analyzed using Statistic Product for Statistical Solutions (SPSS), version 25.0 (SPSS Inc. Chicago, USA). The intention-to-treat principle was applied where computerized multiple imputation methods handled missing data. Descriptive statistics were used to describe the baseline sociodemographic (age, gender, body mass index, and duration of KOA) and clinical outcome characteristics of the participants. For baseline comparability analysis, the one-way analysis of variance was applied to examine the effects of these factors as dependent variables. Further, the analysis of covariance (ANCOVA) was used to evaluate pre-post intervention differences in the KOOS, VAS, and TUG scores between the two groups. All statistical significances were set at *P* < 0.05, and the ES was included to support relevant findings. The ES of each variable was examined using Cohen’s d for between-group differences (0.2, 0.5, and 0.8 as small, medium, and large effects, respectively).^35^

## Results

###  Participant characteristics

**Figure 1:**
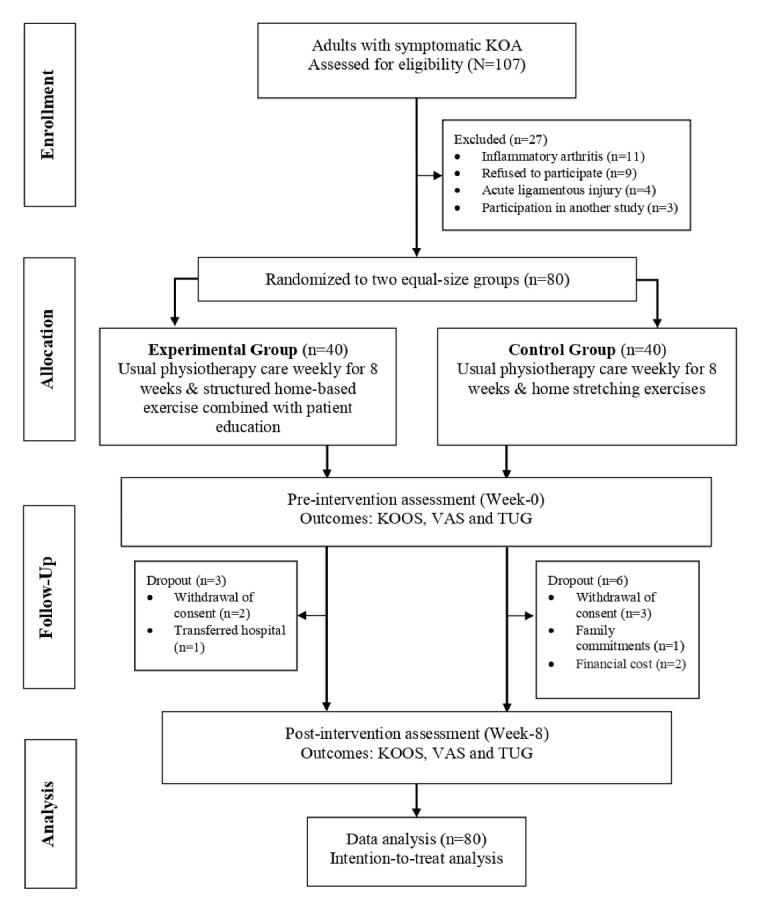
The CONSORT flow diagram. *Note*. KOA: Knee osteoarthritis; KOOS: Knee injury and osteoarthritis outcome score; TUG: Time up- and-go test; VAS: Visual analogue scale.

 The data collection process (participant screening, recruitment, intervention, and outcomes assessments) was completed from June 2017 until January 2022. One hundred and seven adults of both genders with symptomatic KOA were initially screened for eligibility at the Physiotherapy Department of Universiti Kebangsaan Malaysia and Universiti Malaya Medical Centre. Out of this number, 27 cases did not meet the inclusion criteria since they presented with inflammatory arthritis such as rheumatoid arthritis and systemic lupus erythematosus (n = 11), declined to participate (n = 9), had acute ligamentous knee injuries (n = 4), and had recently participated in another interventional study (n = 3). Eighty patients agreed to participate in this study and were randomized into the experimental (19 men and 21 women) and control (15 men and 25 women) groups (Figure 1). The mean ± standard deviation (SD) for age, body mass index (BMI), and the duration of KOA in the experimental (n = 40) and control (n = 40) groups were 65.30 ± 6.90 years, 28.80 ± 3.40 kg/m^2^, 23.00 ± 16.10 months, and 65.60 ± 8.70 years, 26.90 ± 4.40 kg/m^2^, 32.70 ± 31.30 months, respectively. At baseline, there were no significant differences in participants’ demographic variables (age, BMI, and duration of illness) between the two groups. Moreover, no significant differences were found in outcome variables (KOOS, pain VAS, and TUG) measured at baseline between the experimental and control groups. The baseline sociodemographic and clinical outcome characteristics of the participants in both groups are provided in Table 2.

**Table 2:** Baseline sociodemographic characteristics and clinical outcomes of the participants

**Variables**	**Experimental (n=40)**		**Control (n=40)**		***P*** ** values**
	**Mean**	**SD**	**Mean**	**SD**	
Age (y)	65.27	6.86	65.60	8.70	0.919
Body mass index (kg/m^2^)	28.83	3.38	26.86	4.37	0.226
Duration of illness (months)	23.00	16.11	32.73	31.33	0.371
KOOS (Total score)	63.66	9.25	67.98	19.81	0.216
VAS	4.65	1.56	5.10	1.37	0.175
TUG (time, second)	11.57	1.49	11.16	2.68	0.406

*Note*. SD: Standard deviation; KOOS: Knee injury and osteoarthritis outcome score; TUG: Timed up-and-go test; VAS: Visual analogue scale.

 Out of eighty participants, seventy-one (37 experimental and 34 control) completed the eight weeks of intervention and pre-post assessments, representing an adherence rate of 88.9%. At the end of the study, participants’ adherence to the prescribed home exercises (experimental group: Structured HBE and control group: Home stretching) was evaluated based on the average number of exercises performed per week (self-recorded in the training diary). There were no significant differences in the number of home exercise sessions performed between the experimental (2.68 ± 0.87) and control (3.00 ± 1.07) groups (*P* = 0.167). The most frequently reported reasons for non-adherence were the lack of free time and pain restriction. The mean ± SD score changes in the primary and secondary outcomes at baseline (week 0) and post-intervention (week 8) are presented in Table 3.

**Table 3:** Changes in the KOOS, VAS, and TUG scores pre-post intervention

**Outcomes**	**Experimental (n=40)**		**Control (n=40)**		**Between-group (ANCOVA)**		
	**Mean**	**SD**	**Mean**	**SD**	***P *** **value**	***np***^2^	**Effect size**
KOOS (total score)					0.001	0.81	0.66
Pre	63.66	9.25	7.98	19.81			
Post	79.94	5.55	70.30	19.90			
KOOS-symptoms					0.001	0.637	0.746
Pre	8.10	4.24	8.01	5.25			
Post	4.74	2.59	7.79	5.17			
KOOS-pain					0.001	0.749	0.665
Pre	11.72	3.29	11.36	5.90			
Post	7.89	3.25	11.19	6.22			
KOOS-ADL					0.001	0.683	0.191
Pre	52.01	3.62	54.20	8.73			
Post	56.85	3.35	55.60	8.62			
KOOS-sports					0.001	0.53	1.57
Pre	9.14	8.87	7.49	6.59			
Post	22.58	11.28	7.88	6.85			
KOOS-QOL					0.001	0.558	0.911
Pre	9.83	2.55	9.41	3.50			
Post	12.52	2.01	9.37	4.46			
VAS					0.001	0.722	2.633
Pre	4.65	1.56	5.10	1.37			
Post	2.35	0.77	4.82	1.08			
TUG (time, second)					0.001	0.71	1.019
Pre	11.57	1.49	11.16	2.68			
Post	9.59	1.22	11.31	2.05			

*Note*. SD: Standard deviation; ADL: Activities of daily living; KOOS: Knee injury and osteoarthritis outcome score; QOL: Quality of life; TUG: Timed up- and-go test; VAS: Visual analogue scale; ANCOVA: Analysis of covariance; *np*^2^, partial eta squared.

###  Changes in disability level: KOOS

**Figure 2:**
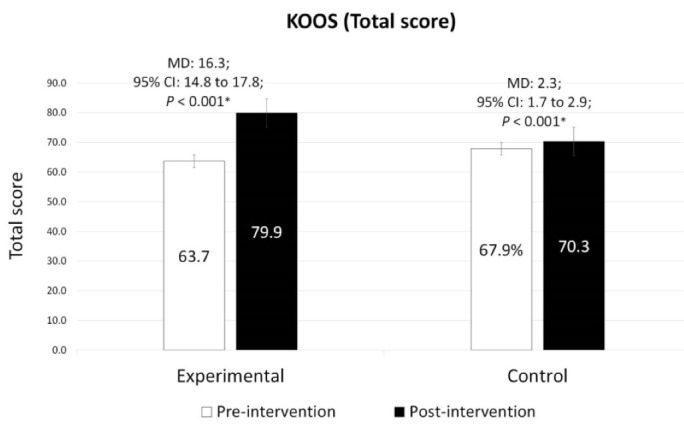
Changes in the KOOS total score pre-post intervention. *Note*. KOOS: Knee injury and osteoarthritis outcome score. Significant within-group differences (*P* < 0.05).

 The ANCOVA for KOOS scores showed significant differences between the two groups (experimental and control) pre-post intervention (Table 3); the differences were related to KOOS total score (MD: 13.62, 95% CI: 12.12 to 15.12, *P* < 0.001), symptoms (MD: -3.12, 95% CI: -3.66 to -2.59, *P* < 0.001), pain (MD: -3.65, 95% CI: -4.14 to -3.18, *P* < 0.001), ADL (MD: 3.35, 95% CI: 2.83 to 3.86, *P* < 0.001), sports (MD: 13.23, 95% CI: 10.40 to 16.06, *P* < 0.001), and QOL subscales (MD: 2.71, 95% CI: 2.16 to 3.26; *P* < 0.001). The within-group comparison demonstrated that the experimental group represented significant improvements in the KOOS total score and each of the subscales (*P* < 0.001, Figure 2) compared to the baseline. On the other hand, only the KOOS total score (*P* < 0.001), ADL subscale (*P* < 0.001), and sports subscale (*P* = 0.042) were significantly improved for the control in comparison to the baseline. Likewise, the percentage of improvement for KOOS (the total score) based on the mean differences of pre-post scores was higher in the experimental group (25.6%) compared to the control (3.4%). Furthermore, Cohen’s d analysis indicated a medium (d = 0.66) to large (d = 2.63) ES between the two groups.

###  Changes in knee pain: VAS

**Figure 3:**
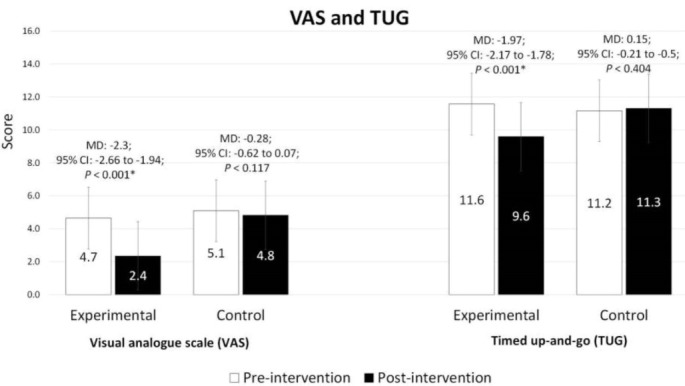
Changes in the VAS and TUG scores pre-post intervention. *Note*. TUG: Time up-and-go test; VAS: Visual Analogue Scale. Significant within-group differences (*P* < 0.05).

 The analysis revealed significant between-group differences for pain VAS scores pre-post intervention (MD: -2.29, 95% CI: -2.60 to -1.96, *P* < 0.001). The within-group comparison demonstrated a significant decrease in pain VAS scores in the experimental group compared to baseline (MD = -2.3,, 95% CI: -2.66 to -1.94, *P* < 0.001, Figure 3). On the contrary, no significant changes of pre-post pain VAS evaluation were found in the control group. A higher percentage of VAS score reduction was observed in the experimental group (-49.5%) in comparison to the control group (-5.4%). A large (d = 0.63) ES was also observed between the two groups based on Cohen’s d analysis.

###  Changes in functional mobility: TUG Test

 The analysis of TUG scores showed significant between-group differences pre-post intervention (MD: -2.01, 95% CI: -2.30 to -1.72, *P* < 0.001). Compared to the baseline, within-group comparison indicated a significant decrease in TUG scores only in the experimental group (MD = -1.97, 95% CI: -2.17 to -1.78, *P* < 0.001). Similarly, the percentage of reduction was higher in the experimental (-17.0%) compared to the control (1.3%) pre-post assessments. Based on Cohen’s d analysis, a large (d = 1.02) ES was found between the two groups.

## Discussion

 The study aimed to evaluate the effects of a structured HBE + EDU program as an adjunct treatment to the usual physiotherapy care on KOA-related clinical outcomes (pain, functional mobility, and disability level). The study findings revealed that following an eight-week intervention, participants in the experimental group demonstrated significant improvements in all investigated outcomes (KOOS, pain VAS, and TUG) compared to baseline. In addition, group effects were observed for KOOS (22.2%), pain VAS (44.1%), and TUG (15.7%). It seems that the integration of a structured HBE + EDU program as an adjunct treatment to individualized KOA rehabilitation is effective in reducing knee pain and disability while increasing functional mobility.

 Based on the VAS assessment, the prescribed HBE + EDU program in this study improved KOA pain. Typically, a reduction in pain is often associated with an increase in strength, as reported by Chen et al,^19^ where an HBE + EDU program improved quadriceps and hamstring muscles’ strength and dynamic stability,^19^ subsequently reducing the abnormal knee joint pressure (load) and inflammation among KOA patients.^17,19^ It is also worth noting that an eight-week of HBE alone focusing on quadriceps strengthening is comparable to non-steroidal anti-inflammatory drugs in improving KOA symptoms, especially pain as the cardinal indicator.^36^ Additionally, self-management information given to patients (as a booklet) alone has also been shown effective in coping with pain.^3,13^ In contrast, adverse conditions have been reported in KOA patients who had limited knowledge of their conditions, particularly concerning the rehabilitation exercise and pain coping strategies,^15^ and this is further aggravated by misconceptions (fear-avoidance beliefs) which negatively affect self-management decisions, health behaviors, and activity participation.^22^ A combination of HBE + EDU seems to be a viable self-management program for coping with KOA pain.

 Furthermore, higher performance in functional mobility and lower fall risk were recorded in the HBE + EDU group compared to the control based on TUG assessments. These can be attributed to various included exercises,^9,16,37^ specifically stretching, which is effective in increasing the active joint range of motion due to growing stretch tolerance.^17^ To our benefit, the quadriceps and hamstring stretching in this study were adapted from Ponvel et al,^38^ confirming to be an effective exercise in evading the risk of falls among KOA patients.^38^ Further, the prescribed strength exercise which focuses on quadriceps (vastus medialis oblique) and hamstrings could serve as a dynamic stabilizer for knee joints.^37,39^ Although strength was not measured in this study, stronger knee muscles would allow for optimal joint stability and mechanics during gait (e.g., increase in step length and cadence).^7,39^ Apart from exercise, improvements in TUG could also be ascribed to effective self-management of pain, which provides indirect augmentation in physical performance, allowing for a higher HBE dosage.^5,37^ Hence, it is suggested that the higher functional mobility performance in the HBE + EDU group could be accompanied by improvements in knee joint range, stability, balance, and lower extremity strength.^7^

 Meanwhile, a significant decrease in the disability level measured using KOOS (as indicated by reduced knee symptoms and improvements in ADL, sports, and QOL) was also observed in the experimental group compared to the control. Generally, disability in KOA is affected by complex interactions between pain, physical capacity.^5,20^ and psychological factors.^23,40^ For these reasons, improvements in knee pain (VAS) and functional mobility (TUG) are believed to play a contributory role in the reduction of disability levels.^5,20,37^ Psychological factors such as fear of movement among KOA patients have profound effects on physical activity^40^ and QOL,^23^ eventually leading to disability.^23,40^ This is further aggravated by a misconception that knee joints would be further damaged by physical activity, where knee movement should be purportedly avoided to minimize the risk of pain exacerbation.^40^ Fortunately, this fallacy is manageable through targeted psychological and behavioral interventions via patient education^13^ and graded exercises.^3,23^ Therefore, the decrease in the level of disability in the HBE + EDU group could be contributed to better management of pain, improvements in physical capacity, and to a certain extent, psychological factors. Moreover, a training diary^17^ and phone call follow-up (once every two weeks) were performed to improve exercise adherence.^19^ These methods are practicable in promoting exercise compliance, elevating confidence, motivation, and self-discipline.^17,19,29,41^ In addition, frequent communication between therapist and patient increases awareness of the benefits of exercise and makes patients feel more comfortable through KOA rehabilitation.^28^

 Some limitations should be considered in this study. First, given the short study duration (our intervention lasted only eight weeks), we could not determine the long-term effects of the HBE + EDU intervention. Further, the rehabilitation exercises were tailored to individual needs based on physiotherapist assessments, and variations in the usual care received between participants may have also influenced our findings. Furthermore, considering that the KOOS and VAS outcomes were assessed based on self-reported methods, the findings could potentially be confounded by the differences in the subject’s perception (risk of method bias).^42^ Hence, further investigations with a longer study duration, objective measures of knee function, and the evaluation of knowledge and perception (qualitative exploration) are possibly needed to establish relevant evidence on the long-term outcomes of HBE + EDU in KOA.

**Highlights**

- Home-based exercise and patient education (HBE + EDU) is a useful adjunct to usual knee osteoarthritis (KOA) rehabilitation
- The HBE + EDU combination reduced knee pain and disability while increasing functional mobility
- HBE enhanced the dosage of exercise therapy
- Patient education promotes self-management skills

## Conclusion

 Overall, it was found that integrating the HBE + EDU program into usual physiotherapy care could effectively reduce knee pain and disability and increase functional mobility among adults with KOA. The use of HBE + EDU is not meant to substitute the current KOA management; on the other hand, it can be employed as a part of a home strategy to promote self-management skills and boost the dosage of exercise therapy for optimal clinical outcomes. Therefore, this study suggests a combination of a structured HBE and EDU program to be considered as part of KOA’s mainstream management.

## Acknowledgements

 The authors thank the participants and management of the Physiotherapy Department at the Universiti Kebangsaan Malaysia and Universiti Malaya Medical Centre for their participation and assistance in this study.

## Competing Interests

 The authors reported no potential conflict of interest.

## Funding

 The research was partly supported by the PV061-2022 PPRN-BTL-UM grant.
